# BiocMAP: a Bioconductor-friendly, GPU-accelerated pipeline for bisulfite-sequencing data

**DOI:** 10.1186/s12859-023-05461-3

**Published:** 2023-09-13

**Authors:** Nicholas J. Eagles, Richard Wilton, Andrew E. Jaffe, Leonardo Collado-Torres

**Affiliations:** 1 Lieber Institute for Brain Development, Johns Hopkins Medical Campus, Baltimore, 21205 USA; 2 Department of Physics and Astronomy, Johns Hopkins University, Baltimore, 21218 USA

**Keywords:** WGBS, Bioconductor, Pipeline, Arioc

## Abstract

**Background:**

Bisulfite sequencing is a powerful tool for profiling genomic methylation, an epigenetic modification critical in the understanding of cancer, psychiatric disorders, and many other conditions. Raw data generated by whole genome bisulfite sequencing (WGBS) requires several computational steps before it is ready for statistical analysis, and particular care is required to process data in a timely and memory-efficient manner. Alignment to a reference genome is one of the most computationally demanding steps in a WGBS workflow, taking several hours or even days with commonly used WGBS-specific alignment software. This naturally motivates the creation of computational workflows that can utilize GPU-based alignment software to greatly speed up the bottleneck step. In addition, WGBS produces raw data that is large and often unwieldy; a lack of memory-efficient representation of data by existing pipelines renders WGBS impractical or impossible to many researchers.

**Results:**

We present BiocMAP, a Bioconductor-friendly methylation analysis pipeline consisting of two modules, to address the above concerns. The first module performs computationally-intensive read alignment using *Arioc*, a GPU-accelerated short-read aligner. Since GPUs are not always available on the same computing environments where traditional CPU-based analyses are convenient, the second module may be run in a GPU-free environment. This module extracts and merges DNA methylation proportions—the fractions of methylated cytosines across all cells in a sample at a given genomic site. Bioconductor-based output objects in R utilize an on-disk data representation to drastically reduce required main memory and make WGBS projects computationally feasible to more researchers.

**Conclusions:**

BiocMAP is implemented using Nextflow and available at http://research.libd.org/BiocMAP/. To enable reproducible analysis across a variety of typical computing environments, BiocMAP can be containerized with Docker or Singularity, and executed locally or with the SLURM or SGE scheduling engines. By providing Bioconductor objects, BiocMAP’s output can be integrated with powerful analytical open source software for analyzing methylation data.

**Supplementary Information:**

The online version contains supplementary material available at 10.1186/s12859-023-05461-3.

## Background

The genome of many organisms is more than just a sequence of four nucleotides. These nucleotides can be chemically modified, and a common modification is the methylation of cytosines [1], which was discovered in mammals as early as DNA itself [2]. The percent of methylated cytosines was first measured across a significant portion of the human genome using methylation arrays [3]. With the advent of whole genome sequencing, whole genome bisulfite sequencing (WGBS) became a reality, allowing researchers to study methylated cytosines in different contexts (CpG, CpH) [4] and experimental settings [3]. However, population-scale studies have generally been limited to microarrays due to complexities in sample pre-processing required for WGBS. While methylation typically occurs primarily at cytosines in CpG context in almost all cell and tissue types, CpH-context methylation is present and plays a significant role in the brain [5]. In contrast to microarrays, WGBS captures CpH methylation and novel CpG loci. Furthermore, a study by Perzell Mandell et al. [6] exploring age and sex-associated methylation differences in human dorsolateral prefrontal cortex samples found that more than 97% of significant CpG loci were unmeasured by microarrays.

Raw sequencing reads require several computational processing steps to produce DNA methylation proportions, a feature ready for statistical analysis. Among the most computationally demanding steps is alignment of reads to a reference genome, where software must consider alignments across reference sequences from 4 methylation states: methylated and unmethylated cytosines under directional and non-directional library preparation protocols. With currently available CPUs, alignment software such as *Bismark* [7] may require hours or days to align a single WGBS sample. The *Arioc* [8] aligner, which uses GPU acceleration to compute WGBS alignments, achieves processing speeds that are an order of magnitude faster without sacrificing accuracy or sensitivity. This provides a natural motivation for the implementation of a workflow that can use GPUs for alignment but CPUs for remaining processing steps. However, the use of GPUs for non-graphics-related tasks is still in its infancy, and GPU resources are sometimes not available on the same computing clusters where traditional CPU and memory resources are abundant.

We introduce BiocMAP [9], a **Bioc**onductor-friendly **M**ethylation **A**nalysis **P**ipeline for processing bisulfite-sequencing data into analysis-ready R [10] objects. As *bsseq* [11] objects, BiocMAP outputs extend the popular *SummarizedExperiment* [12] format and are readily analyzable with a number of Bioconductor [13] R [10] packages, making WGBS research convenient for more of the computational genomics community. BiocMAP is split into two modules that can be executed in different computing environments; this can allow a researcher to align samples in a computing environment with ample GPU resources, but perform “methylation extraction”—calculating the fraction of methylated cytosines at a given genomic site—and remaining processing steps in an environment with more CPUs and memory. BiocMAP is built using Nextflow [14], a popular workflow-management framework enabling simple configuration, effective parallelization, and straightforward support of multiple computing environments. Small edits to configuration files allow any Nextflow workflow to run with Sun/Son of Grid Engine (SGE) or Simple Linux Utility for Resource Management (SLURM) job schedulers, in cloud environments like Google Cloud or Amazon Web Services, and running pipeline components inside Docker or Singularity containers.

## Results

### Overview

**Fig. 1:**
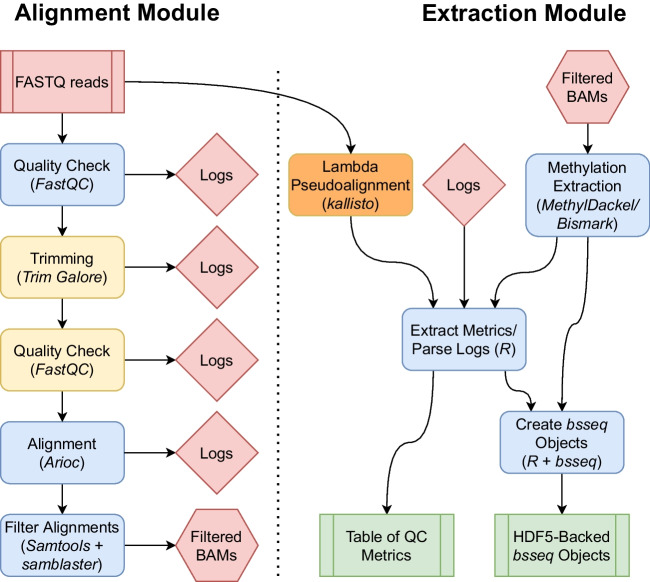
BiocMAP workflow overview. Diagram representing the conceptual workflow traversed by BiocMAP. The red box indicates the FASTQ files are inputs to the pipeline; green coloring denotes major output files from the pipeline; the remaining boxes represent computational steps. Yellow and orange-colored steps are optional or not always performed; for example, lambda pseudoalignment is an optional step intended for experiments with spike-ins of the lambda bacteriophage. Finally, blue-colored steps are ordinary processes which occur on every pipeline execution. Depending on the available high performance computing (HPC) systems, both modules can be run sequentially on a HPC system with both GPUs and CPUs, or the alignment module can be run on a GPU-powered HPC system, then files transferred to a CPU-based HPC system as well as updating file paths on the *rules.txt* file (dotted line), before running the extraction module on the CPU-based HPC system

The BiocMAP workflow consists of a set of two modules—alignment and extraction, which together process raw WGBS reads in FASTQ format into Bioconductor-friendly [13] R [10] objects containing DNA methylation proportions essentially as a cytosine-by-sample matrix (Fig. 1). In the first alignment module, an initial quality check is performed with *FastQC* [15], after which samples are trimmed with *Trim Galore!* [16], aligned to a reference genome with *Arioc* [8], and low-quality or duplicate mappings are filtered out.

**Fig. 2:**
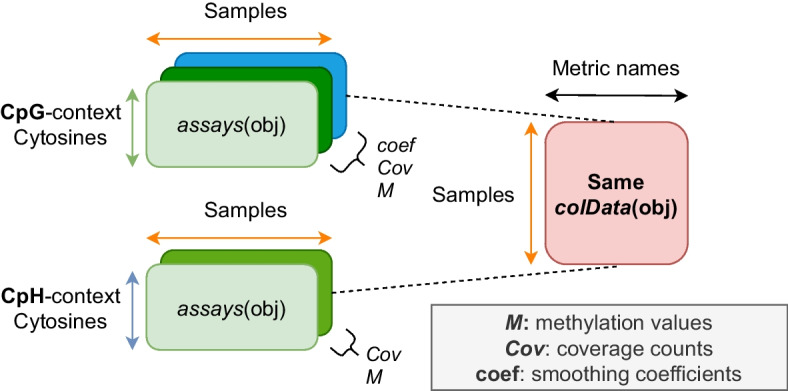
*bsseq* output objects. The major outputs from the extraction module are R objects from the *bsseq* Bioconductor package, which contain methylation proportion and coverage information at all cytosine loci in the human genome. *bsseq* extends the *SummarizedExperiment* class, which provides a general and popular format for storing genomics data and is memory efficient thanks to the *HDF5Array* backend. Two *bsseq* objects are produced, with one object containing cytosine sites in CpG context, and the other containing the remaining CpH loci

In the second extraction module, DNA methylation proportion extraction is performed within each sample using *MethylDackel* [17] or optionally *Bismark* [7], and the results are aggregated across samples into a pair of *bsseq* [11] R [10] objects for easy integration with a number of Bioconductor [13] packages to facilitate downstream statistical analyses. Some examples include *limma* [18] for linear modeling, *Borealis* [19] for outlier detection, and *MethCP* [20] and *DMRcate* [21] for finding differentially methylated regions. The first *bsseq* object contains counts of methylated and unmethylated cytosines in CpG context across the entire reference genome, while the second object contains any additional cytosines in CpH context, when relevant (Fig. 2). A summary table is also produced, compiling together metrics and statistics from trimming, alignment, and methylation extraction for each sample (Additional file 1: S1). Examples of information gathered include percent of reads concordantly aligned and percent of reads trimmed. This allows researchers to control for potential covariates and unintended sources of variation when performing downstream statistical analyses such as the identification of differentially methylated regions (DMRs) [11].

### Application

**Fig. 3:**
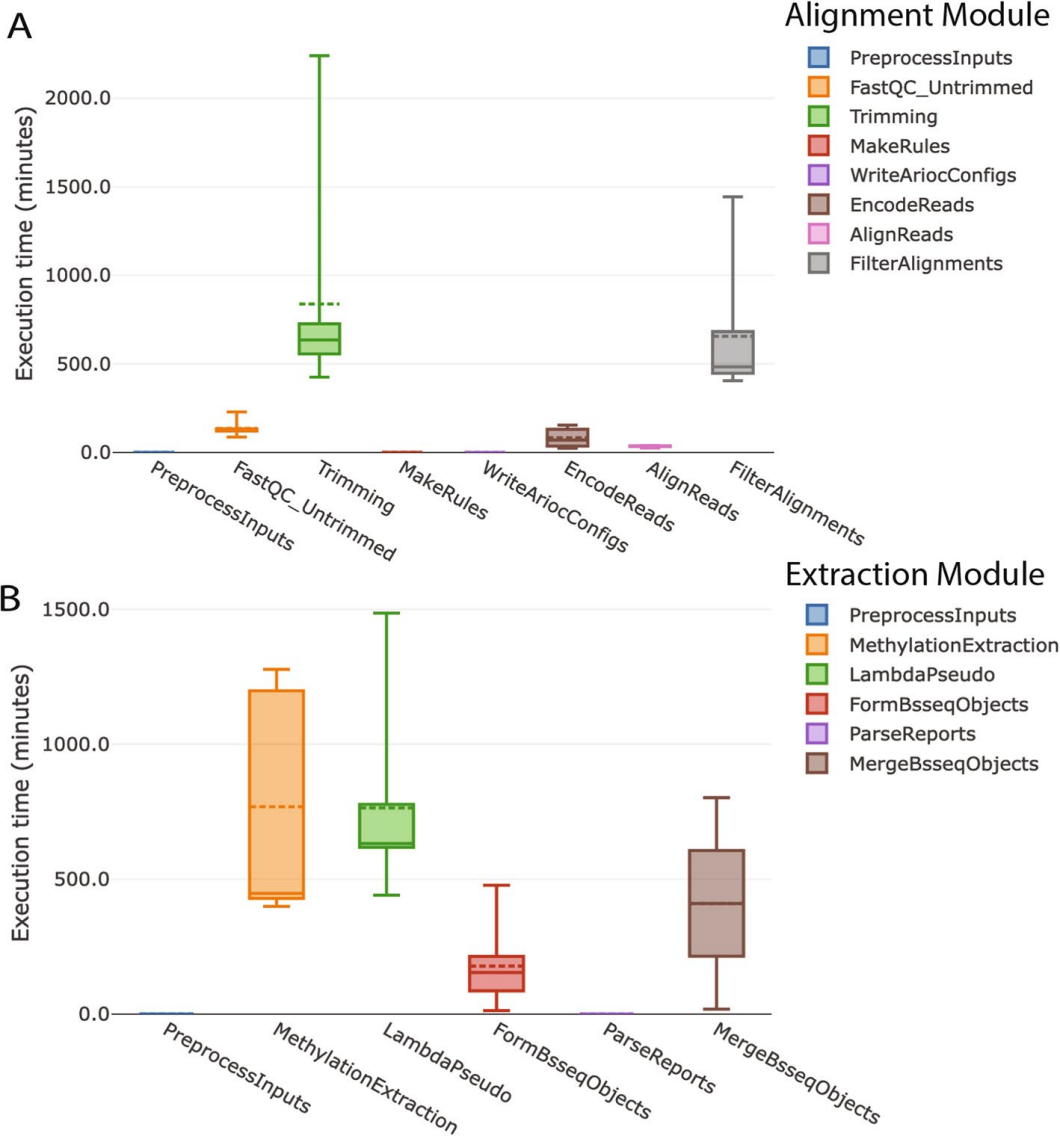
Process run-times from combined execution reports. Wallclock run-times for each process in the alignment and extraction BiocMAP modules, (**A**) and (**B**) respectively, are plotted for the 32-sample subset of the Price et. al dataset [22]. Individual boxplots for each module, combined here for illustration purposes, are one several plot types included in an HTML execution report generated by including the *-with-report* command-line option to BiocMAP, as made possible through the Nextflow [14] framework. A given process, or computational step, in the BiocMAP workflow may be executed for one of more samples in the dataset; boxplots here summarize the distribution of run-times across all executions of each process type

To demonstrate how BiocMAP outputs might be used to perform statistical analysis and visualization on real WGBS data (Additional file 2), we used a publicly available dataset and illustrate an example analysis [9]. The dataset used includes 32 human brain dorsolateral prefrontal cortex (DLPFC) samples spanning developmental years from postnatal up to 23 years of age [22]. NeuN-based fluorescence-activated nuclear sorting was used to produce 8 glial and 24 neuronal samples from the homogenate DLPFC tissue. Prenatal samples present in Price et al. [5] were excluded for this analysis. As CpH methylation is known to be crucially involved in age-related neuronal development [5], this dataset exemplifies a practical research use-case for BiocMAP analysis. As a whole-genome human dataset, it also reflects the potentially large scale of data possible to process with BiocMAP. The first module was run with the default *jhpce* configuration, which runs at most 40 concurrent Nextflow processes, performs alignment with 3 NVIDIA A100 80 GB GPUs, with virtual memory peaking at 276 GB during alignment. The second module was also run with *jhpce* defaults and 35 concurrent processes, peaking at 142 GB of virtual memory usage. Resulting R objects load in 23 GB of memory. Execution times for each computational step in BiocMAP with this dataset were recorded and provide a guideline for other datasets (Fig. 3). The execution time boxplots that can be generated as part of a Nextflow [14] “execution report”, produced by including the *-with-report* command-line option to the appropriate BiocMAP execution script (Additional file 1: S2).

**Fig. 4:**
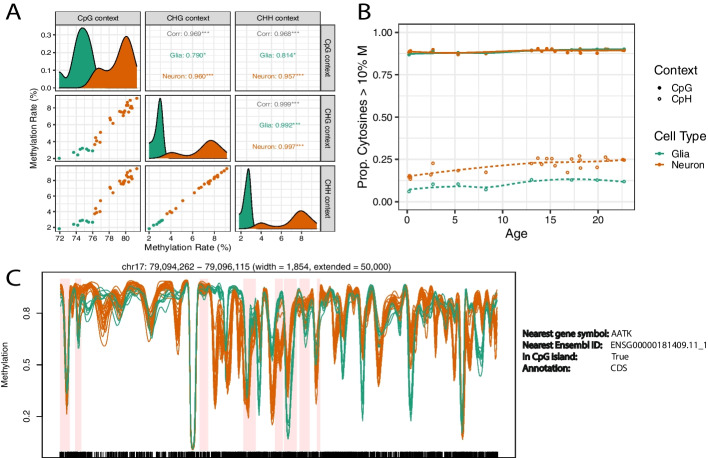
Visualization of BiocMAP outputs on an Example Dataset. **A** Comparison of average methylation rate by trinucleotide context and cell type, showing significant correlation between contexts and similar methylation distributions with different means by cell type. Glia have lower correlation with CpG and a lower methylation rate than neurons. **B** Proportion of highly methylated cytosines across age by cell type and trinucleotide context. Here it can be seen that neuronal CpH methylation appears to generally increase with age, with no obvious association with age in other combinations of cell type and context. **C** Genomic region containing differential methylation between neurons and glia. Orange and green methylation curves represent neuronal and glial samples, respectively. Windows highlighted in light red show differentially methylated regions (DMRs) determined in the Price et al. manuscript [5]

In the example analysis, we show how to attach external sample metadata to the *bsseq* [11] objects and produce several exploratory plots (Additional file 2). For example, we compared the estimated bisulfite-conversion rate (produced by BiocMAP) across neuronal and glial samples (provided by the dataset metadata; Additional file 2). We examined the relationship between methylation contexts (CpG, CHG, CHH), which shows higher methylation rates for neurons compared to glia as well as higher correlation between the methylation rate in CpG context against CHH or CHG context in neurons versus glia (Fig. 4A). The proportion of cytosines with methylation higher than 10% is not significantly different under the CpG context, but is on the CpH context (CHH or CHG) when comparing neurons versus glia (Fig. 4B). This proportion doesn’t change across ages 0 to 23. The original study describing this dataset identified differentially methylated regions (DMRs) between cell types [5]. By providing R/Bioconductor objects, BiocMAP’s outputs can easily be integrated with other R/Bioconductor packages that provide statistical and visualization methods. For example, DMRs can be visualized with *bsseq* [11] (Fig. 4C).

### Benchmark

**Fig. 5:**
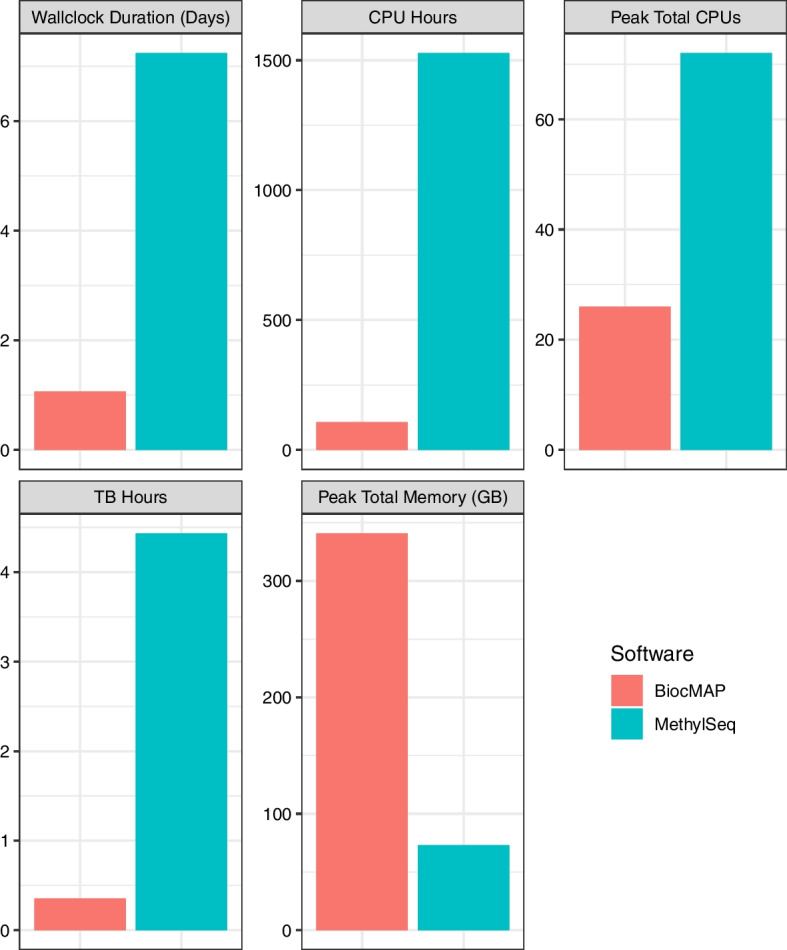
Benchmark results. **A** Total wallclock duration of each pipeline. BiocMAP completes in roughly 1 day, not including downtime between the first and second module, while nf-core/methylseq completes in roughly 7 days. **B** Total CPU utilization by pipeline, in CPU hours. nf-core/methylseq uses more than 14 times the CPU resources as BiocMAP, at 1527 CPU hours. BiocMAP uses less than 8.7 GPU hours total (not shown) during alignment, suggesting the greater CPU usage of nf-core/methylseq is not explained by BiocMAP’s use of GPUs. **C** Maximal concurrent CPU usage at any point during pipeline execution. BiocMAP utilized 26 CPUs at its most demanding moment, while nf-core/methylseq used 72. Concurrent CPU usage is configurable, but was designed to be unconstrained during this benchmark. **D** Total memory usage by each pipeline in TB-hours. nf-core/methylseq used approximately 12.5 times more memory hours than BiocMAP over the full course of execution. **E** Maximal concurrent memory usage in GB across all processes, at any point during pipeline execution. BiocMAP used 341 GB of memory, approximately 4.7 times more than nf-core/methylseq, at each of their most memory-intensive moments. Note that concurrent memory usage is configurable, but was intentionally unconstrained for this benchmark

To quantify BiocMAP’s performance, we selected a 4-sample subset of the Price et. al. dataset [22] and ran BiocMAP in its entirety along with a popular and functionally similar WGBS pipeline, nf-core/methylseq [23]. We found that BiocMAP is considerably faster while using significantly less CPU hours and memory (total TB hours) (Fig. 5). These two metrics are the ones typically quantified for billing purposes on high performance computing environments, thus BiocMAP can reduce computational expenses associated with pre-processing of WGBS data, in addition to time gains. On the other hand, BiocMAP was significantly more demanding at its peak in terms of total concurrent memory usage, at 341 GB, compared with nf-core/methylseq’s 73 GB. During the benchmark, each tool was allowed 40 concurrent Nextflow processes and unlimited CPU and memory resources (*Methods: Benchmark*). Given these loose constraints, BiocMAP aggressively parallelizes steps and consequently utilizes a much larger peak memory than nf-core/methylseq, despite an overall  12.6-fold lower overall memory utilized (by total TB hours). The most demanding step in BiocMAP requests 180 GB by default, imposing a practical minimum of 180 GB for a system to run BiocMAP from start to end. Moreover, this value can generally be reduced in BiocMAP configuration files (*Methods: Configuration*) for datasets consisting of less than about 400 samples.

## Methods

### Overview

In the first alignment module, FASTQ files are checked with *FastQC* 0.11.8 [15], and by default samples that fail the “Adapter Content” metric are trimmed using *Trim Galore!* 0.6.6 [16] (see *Methods: Configuration* for alternative trimming options). The resulting FASTQ files are aligned to a reference genome using the GPU-based aligner *Arioc* 1.43 [8], to produce alignments in SAM format [24]. Using *SAMtools* 1.10 [24], alignments are filtered such that only primary alignments with $MAPQ \ge 5$ are kept, duplicate reads are dropped using *SAMBLASTER* 0.1.26 [25], and finally the result is coordinate-sorted, indexed and stored in BAM format via *SAMtools*.

In the second extraction module, methylation extraction is performed with *MethylDackel* 0.5.2 [17] or optionally *Bismark* 0.23.0 [7] on alignments from the alignment module. By default, a BAM file and corresponding index are expected as input to *MethylDackel*, while just a SAM or BAM file is required if *Bismark* is to be used. For experiments using spike-ins of the lambda bacteriophage, we use a pseudo-alignment-based approach to infer bisulfite-conversion efficiency for each sample. In particular, we prepare two “versions” of the lambda genome: the original and a copy where each cytosine is replaced with a thymine (Availability of data and materials). The latter version represents “in-silico bisulfite conversion” of the original genome, where we assume this original is completely unmethylated. Using *kallisto* 0.46.1 [26], we count the number of reads aligned to each version of the lambda genome, and call these counts *o* and *b* for the original and bisulfite-converted versions, respectively. We define the bisulfite conversion efficiency rate *e* with the following ratio: $e=\frac{b}{o + b}$. This contrasts with the more conventional approach [27], which involves directly aligning reads to the lambda reference genome and comparing cytosine and thymine counts on a single strand. In our own tests, we have found our pseudo-alignment-based approach to be sufficiently concordant with the conventional method, while requiring just a fraction of the computational time. The Bioconductor package *bsseq* [11] is used to gather methylation information into *SummarizedExperiment*-based [12] objects. The final result is a pair of *bsseq* objects, each of which contains all samples in the experiment as columns (Fig. 2). One object contains all cytosines in the genome observed in CpG context, represented as rows, while the other contains the remaining cytosines in CpH context (Fig. 2). Various metrics from *FastQC* [15], trimming, alignment, methylation extraction, and lambda pseudoalignment, if applicable, are aggregated into the *colData()* slot of each object (Additional file 1: S1).

### Configuration

A researcher typically must modify one or two types of files in BiocMAP: the execution script and optionally, a configuration file. The execution script contains the *nextflow* command [14] and major experiment-specific options, which together invoke one module in BiocMAP on a particular platform. Execution script templates are provided for each module in BiocMAP (“alignment” and “extraction”) and platform, which includes local Linux machines and systems managed by the Sun/Son of Grid Engine (SGE) or Simple Linux Utility for Resource Management (SLURM) job schedulers (Additional file 1: S2). Additional job management systems are supported by Nextflow [14].

Required arguments in each execution script include “reference” and “sample”. “reference” may take values “hg38” or “hg19”, corresponding to the reference human genome to which samples should be aligned. The mouse reference genome “mm10” is also supported. “sample” may take values “single” or “paired”, referring to whether samples are single-end or paired-end. Several other options may be configured in the execution script. For example, the “input” argument takes the directory including *samples.manifest* for the alignment module, and the directory containing *rules.txt* for the extraction module (Additional file 1: S3). The optional “trim_mode” argument in the alignment module may take values “skip”, “adaptive”, or “force”, allowing the user to avoid trimming any samples, trim samples whose “Adapter Content” metric from *FastQC* [15] is “FAIL”, or trim all samples, respectively.

### Inputs

Both the alignment and extraction modules require a file called *samples.manifest* as input; this file is identical to those used in SPEAQeasy [28], thus providing a common file for describing input samples in both RNA-seq and WGBS projects. Briefly, *samples.manifest* is a tab-delimited plain-text file containing the absolute paths to each FASTQ file in the experiment, and associating files with a sample identifier. For compatibility with a previous format, optional MD5 sums can be associated with each FASTQ file. FASTQ files may optionally be compressed using gzip, so “.fq”, “.fastq”, “.fq.gz”, and “.fastq.gz” are all accepted file extensions. A researcher can specify that any combination of files be merged and treated as a single sample, simply by using the same sample ID in each line of FASTQ files to combine. This allows for simple management of the common case where a single biological sample is sequenced across several sequencing lanes and thus produces several files.

The extraction module makes use of the same *samples.manifest* file as the alignment module, as well as an additional file called *rules.txt* (Additional file 1: S3). The purpose of this file is to direct BiocMAP to each of the sets of outputs produced from the alignment module, which may not necessarily have been produced on the same high performance computing (HPC) system. From the alignment module, a researcher will have produced SAM or BAM files from alignment, as well as logs from quality checking, trimming, and alignment. The extraction module requires that a researcher create the *rules.txt* file, and point to the directory containing it via the *–input* option in the main submission script. *rules.txt* is a plain-text file where each line consists of a key-value pair (Additional file 1: S3). To facilitate this process, the alignment module creates a template *rules.txt* file which can be easily updated by the researcher in case file paths change in HPC system used for the extraction module. As an example *rule*, the “sam” key requires the path to each SAM or BAM from alignment as a value. Other keys are “manifest”, “arioc_log”, “xmc_log”, “trim_report”, “fastqc_log_first”, and “fastqc_log_last”, some of which are optional. Because an experiment typically consists of many samples, each value typically refers to multiple paths; including the literal “[id]” in a particular path signals for BiocMAP to replace “[id]” with each sample name to determine the full set of paths. A path can also be written as a glob expression, which is useful whenever a key refers to more than one file per sample; for example, the “sam” key can accept a BAM file and its index for every sample. For more detail about *rules.txt* and properly specifying file paths, see the documentation site (Availability of data and materials).

### Outputs

The final product from running BiocMAP is a pair of *bsseq* R objects, together containing methylation proportion and coverage information at all cytosine loci in the human or mouse genome (Fig. 2). One object contains all cytosines occurring in “CpG” context in the reference genome, while the other object contains the remaining cytosines in “CpH” context. The *bsseq* [11] class extends the *SummarizedExperiment* [12] class, and here rows correspond to cytosines, while columns correspond to samples. Both objects contain the *M* and *Cov* assays, stored as *DelayedArray*s [29] and representing methylation proportions and coverage counts, respectively. We “strand-collapse” CpG loci, which involves combining methylation data from both genomic strands and thus discards strand-specific information. The CpG object is also smoothed using the *BSmooth* [30] pipeline as implemented in the *bsseq* [11] package, yielding an additional assay called *coef*, which contains the smoothing coefficients corresponding to each raw methylation proportion stored in the *M* assay. Computing *coef* is time consuming, which is why for the CpH methylation data BiocMAP does not compute it as typical analyses will first filter the CpH data before computing this matrix. For a typical experiment involving many samples, these objects might occupy tens or even hundreds of gigabytes in memory if loaded in a traditional fashion. To enable working with the objects in a reasonable amount of memory, the assays are HDF5-backed using functionality from the *HDF5Array* [31] package. The HDF5 format is designed to allow direct manipulation of on-disk data as if it were loaded in random access memory (RAM), thus reducing the required RAM by an order of magnitude. The large matrices containing methylation proportions, coverage, and smoothing coefficients are not loaded into memory, and common operations on these matrices are “chunked”, meaning only small pieces of a matrix are loaded into memory (RAM) at a time.

A table of metrics is stored in the *colData()* slot of each object, containing information collected from quality checking, trimming, alignment, methylation extraction, and potentially pseudoalignment to the lambda transcriptome (Additional file 1: S1). These metrics can be used for exploratory data analysis as well as for adjusting for them when performing downstream statistical analyses such as the identification of differentially methylated regions (DMRs). This table is also produced as a standalone R data frame to provide a format that is trivial to load into memory, interactively explore through https://libd.shinyapps.io/shinycsv/ [32], or export to other formats.

In addition to the primary outputs of interest, BiocMAP produces a number of output files from intermediate pipeline steps (Additional file 1: S4).

### Software management

BiocMAP is designed to run on Linux machines, either locally or through the SGE or SLURM job scheduling engines, or other engines supported by Nextflow [14]. We require Java 8 or later to be installed, as well as docker or singularity, based on the user’s preferred installation method. If neither are available, R [10] 4.0 or later and python 3 are required. Finally, an NVIDIA GPU and its corresponding drivers are required. Docker users must also have the NVIDIA container toolkit installed.

BiocMAP makes use of a number of external software tools which must be installed to use the pipeline. We support three different installation methods to accommodate a user’s existing set-up: download of docker images containing software, download of corresponding singularity images, or direct “local” installation of software. All three methods require simply invoking a shell script, followed by the name of the installation method (“docker”, “singularity”, or “local”):
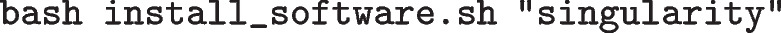


For users of computing clusters, we make the assumption that GPU resources are accessible via a particular queue. Therefore, cluster users must also perform an additional manual step to complete the installation; this involves setting a variable in the appropriate configuration file (Additional file 1: S2) to the name of the queue where GPU(s) are available.
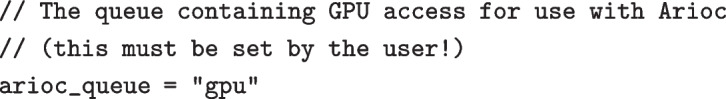


We recommend using the “docker” or “singularity” installation methods, if those tools are available, or the “local” method otherwise. As a Nextflow-based [14] pipeline, BiocMAP is out-of-the-box able to execute individual pipeline components, called *processes*, inside Docker or Singularity containers. These containers provide an exact environment, including the main software, system tools, and other dependencies, so that each BiocMAP process behaves identically on different computing systems. We host every docker image used by BiocMAP on a public Docker Hub repository (Availability of data and materials). In practice, the “singularity” installation mode automatically pulls the required docker images and builds singularity-compatible equivalents to use at run-time.

Alternatively, a researcher may use the “local” installation mode, which builds individual software tools from source when possible or downloads pre-compiled binaries otherwise. Since each piece of software is installed to a local directory and not globally, root permissions are not required for this installation method, and thus might be preferred by some users. However, because this approach tailors the installation to a particular computing environment, it is beyond our capacity to test unlike the “docker” or “singularity” modes, and we thus encourage you to avoid using the “local” mode.

### Annotation

Since BiocMAP performs alignment to a reference genome and can quantify lambda spike-ins [27], it must make use of external reference files. By default, required reference files are automatically pulled from GENCODE [33] (or NCBI for the lambda genome), but a user can also opt to provide their own files instead. The method used by BiocMAP to manage external reference files is nearly identical to that used in SPEAQeasy [28], and we encourage those interested to refer to that manuscript for more details; however, we provide a brief summary here.

When using default annotation from GENCODE [33], the genomes “hg38”, “hg19”, and “mm10” are supported; one of these values must be passed to the $--reference$ option. A researcher may specify the GENCODE version for human or mouse, as appropriate (e.g. “38” or “M27”, respectively). An additional configuration variable called “anno_build” determines if all sequences present in the “primary_assembly” file from GENCODE are kept, or if only canonical reference chromosomes are used for alignment; this corresponds to the values “primary” or “main” that a researcher may select, respectively. BiocMAP only pulls files from GENCODE that have not already been downloaded; after the first execution of the workflow for a given set of settings, it uses a locally cached copy of relevant files. A researcher may manually choose a directory to place annotation files via the command-line option “–annotation [path to directory]”, which enables potentially many users to share a single location for reference files to save disk space and time.

Alternatively, a researcher may provide their own reference genome in FASTA format in place of the automatically managed GENCODE [33] files. In this case, the “–annotation [path to directory]” option signifies the directory containing the provided FASTA file, and the “–custom_anno [label]” option assigns an informative label, or name, that can later be used in place of explicitly providing the genome. Note that the lambda genome is only automatically managed, since it is unlikely a user will need to swap out a different version.

### Test samples

Small test files are provided in the *test* directory of the GitHub repository, for each combination of species (human and mouse) and pairing (single-end and paired-end). These are intended to allow a researcher to quickly verify BiocMAP has properly installed. While human, paired-end files are from the example *AgeNeunSortedWGBS* dataset [5, 22], the remaining files were retrieved from the Sequence Read Archive (SRA) (Additional file 1: S5). All FASTQ files were subsetted to 100,000 reads. A researcher can opt to run the extraction module on test data, without needing to run the alignment module beforehand. Test inputs to the extraction module, which include BAM files, their indices, and logs up through alignment, were generated by running BiocMAP with default settings, with the exception of using *–trim_mode “force”* in place of the default *–trim_mode “adaptive”*.

### AgeNeunSortedWGBS samples

The vignette provided with BiocMAP makes use of a dataset that includes 32 human postnatal dorsolateral prefrontal cortex samples up to 23 years of age [5, 22]. Homogenate postmortem tissue was sorted with NeuN-based fluorescence-activated nuclear sorting to produce 8 glial and 24 neuronal samples. The Price et al. manuscript also included prenatal samples that were excluded for this analysis [5].

*HDF5Array* 1.22.1 [31] is used to load the *bsseq* objects partially into memory, while keeping the *assays()* on disk as *DelayedMatrix* objects from the *DelayedArray* 0.20.0 [29] package. Exploratory plots use *ggplot2* 3.3.5 [34] and the *ggpairs()* function from *GGally* 2.1.2 [35]. Methylation curves at a genomic region with several individual DMRs are explored with *plotRegion()* from *bsseq* 1.30.0 [11]. A total of 42 GB of memory is required to run this analysis, and the analysis completes in 55 min.

### Benchmark

BiocMAP was installed with “jhpce” mode and run with default configuration and command-line options. It was previously run with the same annotation settings on the testing machine, meaning the benchmarking run of BiocMAP excludes one-time steps related to pulling annotation files and encoding for use in Arioc. Because the first module of BiocMAP completed overnight, the second module was not run immediately in succession. The downtime between executions of the first and second modules of BiocMAP was not counted towards computing total wallclock duration (Fig. 5A).

First, a dry run of nf-core/methylseq [23] was performed using the singularity profile and the *GRCh38* genome to install local files, singularity-related dependencies, and cache genome-related files. As its base configuration does not allow for execution on an SGE cluster where computational resources were available, the base configuration was modified. In particular, any memory request was modified to use the “mem_free” and “h_vmem” resources instead of Nextflow’s default of “virtual_free”; this was changed to meet JHPCE usage guidelines. The following segment of code was added to configuration to specify at most 40 concurrent processes, and usage of the SGE job scheduler, settings identical to BiocMAP: 
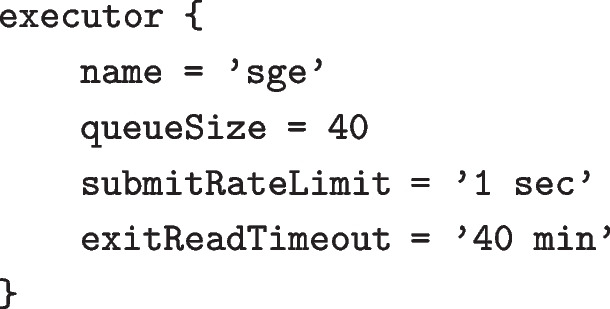


Next, memory requests were raised for *process_single* and *process_low* to 20 G from 6 G and 12 G, respectively. This allowed singularity 3.6.0 to have sufficient memory to run on our machines. Finally, time limits were removed on all processes after exceeding some limits in initial tests. After establishing a successful configuration, nf-core/methylseq was run with otherwise default settings with the singularity profile and *GRCh38* reference genome.

Both BiocMAP and nf-core/methylseq were run on a system with enough RAM and CPUs, such that jobs were not on hold in the SGE queue. In other words, both software tools executed processing steps as soon as previous steps finished, enabling us to compare pipelines by total wallclock time.

## Discussion

Alignment to a reference genome is often the most computationally intensive component of a whole genome bisulfite sequencing (WGBS) data-processing workflow. As a result, workflows with an efficient alignment step can reduce total time required to process a dataset by a significant factor. CPU-based aligners like *Bismark* [7] or the more recent *BS-Seeker3* [36] can process WGBS samples in hours or days, but the GPU-based *Arioc* aligner offers higher alignment speeds than CPU-based aligners while maintaining comparable accuracy [8, 37] (Additional file 3). Furthermore, we demonstrate that the BiocMAP workflow as a whole is significantly faster than comparable CPU-based workflows, making analyses of several-hundred-sample WGBS datasets feasible.

Given the recent introduction of GPUs and limited availability, researchers might not have access to GPUs on their main high performance computing (HPC) environment. HPC systems with GPUs might be under high demand or more expensive to use. Nextflow [14] does not provide functionality for executing some processing steps in one HPC system, transferring files, and resuming executing processes on a second HPC system. For these reasons, BiocMAP was implemented as two separate modules such that processing steps that benefit from the presence of GPUs can be run on HPC systems with GPUs, and the remaining steps can be run on regular CPU-powered HPC systems. Ultimately, if you have access to a HPC system with GPUs, you might prefer to run both modules on such a system. In that situation, BiocMAP’s two modules can be run serially without having to edit the *rules.txt* file that is automatically generated by the alignment module.

While BiocMAP is already likely to align reads quickly through *Arioc* with default settings, researchers are highly encouraged to configure BiocMAP settings to most efficiently use *Arioc* given the available hardware as noted on the documentation website (Availability of data and materials). Most configuration variables used by *Arioc* [8] can be directly edited in the appropriate BiocMAP configuration file (Additional file 1: S2). For example, the *batchSize* BiocMAP configuration variable is passed to the *batchSize* attribute of the *AriocU* or *AriocP* element of the configuration for *AriocU* or *AriocP*, which specifies how many reads *Arioc* can concurrently align per GPU utilized. This is one of many settings that depends on the specifications of the GPU(s) a researcher has available in their HPC system, which can be adjusted to achieve greater throughput. The *max_gpus* BiocMAP configuration variable specifies how many GPUs to use for alignment of each sample, potentially allowing increased parallelism when there is an abundance of GPU resources relative to number of samples in the experiment. A more comprehensive guide to adjusting BiocMAP configuration for a given computing environment is provided as part of the documentation website (Availability of data and materials).

We demonstrated how BiocMAP can be used to process publicly available WGBS data using an example dataset [5, 22]. Additional file 2 shows how you can then load the outputs of BiocMAP and use the data with R and Bioconductor packages such as *bsseq* [11], *ggplot2* [34], and *GGally* [35] to perform exploratory data analysis as well as downstream statistical analyses. In addition, development versions of BiocMAP were used in other peer reviewed publications [6, 38, 39] that have publicly available R code for several downstream analyses.

While we used a dataset of 32 samples to exemplify BiocMAP [5, 22], memory requirements scale roughly linearly with number of samples, with the production of a 600-sample dataset requiring about 200 GB of RAM, despite CpH and CpG cytosines encompassing around half of the genome (depending on the GC content of the genome; private WGBS datasets). Thus BiocMAP is scalable to a sample size larger than most if not all current WGBS datasets. The 200 GB RAM can likely be drastically reduced by future internal BiocMAP updates and can definitely be reduced for any dataset once you apply a filter on the number of reads per cytosine, stored in the *Cov* assay. Despite the potentially large memory requirements for running BiocMAP, loading the output *bsseq* [11] objects requires significantly less memory and is independent of the number of samples in the dataset, thanks to the HDF5 storage backend [31]. Users most likely need around 20–30 GB of RAM to load filtered *bsseq* objects for downstream statistical analyses for the CpH context, while the CpG context object requires less than 1 GB of RAM.

We envision that most users will not be interested in tweaking WGBS processing steps as long as they generate the output in a reasonable amount of time, but instead will want to focus on downstream analyses. We implemented BiocMAP in such a way that it will benefit from community developments in Bioconductor [13]. The main output data container is a *bsseq* [11] object that is an extension of *SummarizedExperiment* [12]. *SummarizedExperiment* itself is the one compatible with low-memory footprint backends such as *HDF5Array* [31]. If *SummarizedExperiment* becomes more efficient, by for example providing a low-memory footprint option for the gene coordinates (*rowRanges()* slot), users of BiocMAP will benefit from the reduction in RAM required to generate and load BiocMAP’s outputs. Similarly, if new R/Bioconductor packages are developed that implement downstream statistical analyses, they will be compatible with BiocMAP’s output objects as they are the central format for DNA methylation data [11, 13]. *zellkonverter* [40] is a Bioconductor package that allows exporting *SingleCellExperiment* R objects to Python. Given that *SingleCellExperiment* is an extension of *SummarizedExperiment*, just like *bsseq*, it seems reasonable to expect that *bsseq* objects will be readable from Python. Given these reasons, we envision that BiocMAP’s users will be able to use the resulting *bsseq* objects with any new methods implemented in R and most likely Python, two of the most widely used programming languages.

Despite the potential for customization within BiocMAP, it is designed to run “out of the box”, without a strict need to make hardware-specific configuration. This enables researchers to focus on their particular analysis questions instead of technical processing details.

## Conclusion

We implemented a whole genome bisulfite sequencing (WGBS) data processing workflow that relies on the GPU-accelerated *Arioc* aligner [8], yet is flexible enough to be used on multiple high performance computing (HPC) systems. The alignment output is further processed and packaged into *bsseq* [11] R/Bioconductor objects that are memory efficient and deeply integrated with the R/Bioconductor open source software ecosystem [13]. Thus BiocMAP will get the data processing job done in a fast and efficient manner for WGBS datasets up to several hundred samples, allowing researchers to focus their attention on exploratory data analysis and downstream statistical analyses. BiocMAP is available and documented at http://research.libd.org/BiocMAP/.

## Availability and requirements

Project name: BiocMAP

Project home page: https://github.com/LieberInstitute/BiocMAP

Operating system(s): Linux

Programming language: Nextflow, R, Groovy

Other requirements: Java 8 or higher, access to NVIDIA GPU(s), Singularity or Docker (recommended)

License: GNU GPLv3

Any restrictions to use by non-academics: N/A

### Supplementary information

**Additional file 1.** Various tables with information about BiocMAP inputs, outputs, test files, and more. This is provided as a multi-sheet excel file, with each sheet described in more detail below. S1: List of output metrics collected by BiocMAP. These are various quantities aggregated from processing steps like *FastQC* [15], trimming, alignment, and methylation extraction. Together they form an R *data.frame* accessible from the file *metrics.rda* and from within the *colData()* of output *bsseq* [11] objects. For paired-end samples, some metrics are computed separately for each mate, in which case metric names are appended with “_R1” and “_R2” to refer to each mate, respectively. S2: BiocMAP execution scripts and associated configuration files. BiocMAP provides several potential files for out-of-the-box functionality on local Linux machines as well as on SLURM or SGE-managed computing clusters. S3: Content of *rules.txt*. Each line of this input file to the extraction BiocMAP module consists of key-value pairs of the form $<key> = <value>$, some of which are required. S4: Intermediate output files. These files are not the main output files of interest from running both modules of BiocMAP, but are generated along the way as byproducts. S5: Sources of test data provided in the BiocMAP repository. Human and mouse single-end and paired-end samples are provided to allow users to quickly verify proper installation of BiocMAP, sourced from SRA or the FlowRNA-WGBS dataset.

**Additional file 2.** Example vignette showing the use of BiocMAP output objects in downstream analysis. This is a PDF file walking through R code and exploratory plots applied on the Price et al. data [22]. This file is also available from the BiocMAP GitHub repository (Availability of data and materials).

**Additional file 3.** Comparison of alignment results between Arioc and Bismark. In 94 postmortem human brain samples from the dorsolateral prefrontal cortex (DLPFC) and hippocampus (HIPPO) [40], Arioc 1.25.2401.18201 [8] maps reads at a consistently higher rate than Bismark 0.19.0 [7], while finding similar methylation rates in different cytosine contexts. Results are colored by brain region and diagnosis (control and schizophrenia), but are fairly uniform across these categories, with the exception of generally higher CHG-context methylation reported by both tools in the DLPFC.

## Data Availability

The BiocMAP software is available from GitHub at https://github.com/LieberInstitute/BiocMAP [9], with documentation at http://research.libd.org/BiocMAP/. The WGBS data used in the example vignette is available from https://www.synapse.org/#!Synapse:syn5842535 [5]. The original version of the lambda genome is available at ftp://ftp.ncbi.nlm.nih.gov/genomes/all/GCA/000/840/245/GCA_000840245.1_ViralProj14204/GCA_000840245.1_ViralProj14204_genomic.fna.gz. Docker images required for BiocMAP are hosted at https://hub.docker.com/orgs/libddocker/repositories.

## Abbreviations

BiocMAP: Bioconductor-friendly Nextflow-based methylation analysis pipeline
CpG: Cytosine preceding a guanine (methylation context)
CpH: Cytosine preceding a nucleotide other than guanine (methylation context)
CHG: Cytosine followed by non-guanine nucleotide, then guanine (methylation context)
CHH: Cytosine followed by two nucleotides other than guanine (methylation context)
CPU: Central processing unit
CUDA: Compute unified device architecture
DMR: Differentially methylated region
GPU: Graphics processing unit
HPC: High performance computing
NCBI: National Center for Biotechnology Information
RAM: Random access memory
SGE: Sun grid engine or son of grid engine
SLURM: Simple Linux Utility for Resource Management
SRA: Sequence read archive
WGBS: Whole genome bisulfite sequencing
