# Supplementary figures and images for: BiocMAP: a Bioconductor-friendly, GPU-accelerated pipeline for bisulfite-sequencing data
Source: BMC Bioinformatics. 2023 Sep 13;24:340. doi: 10.1186/s12859-023-05461-3 (PMC10498615; doi:10.1186/s12859-023-05461-3)

# Mapping Efficiency (%)

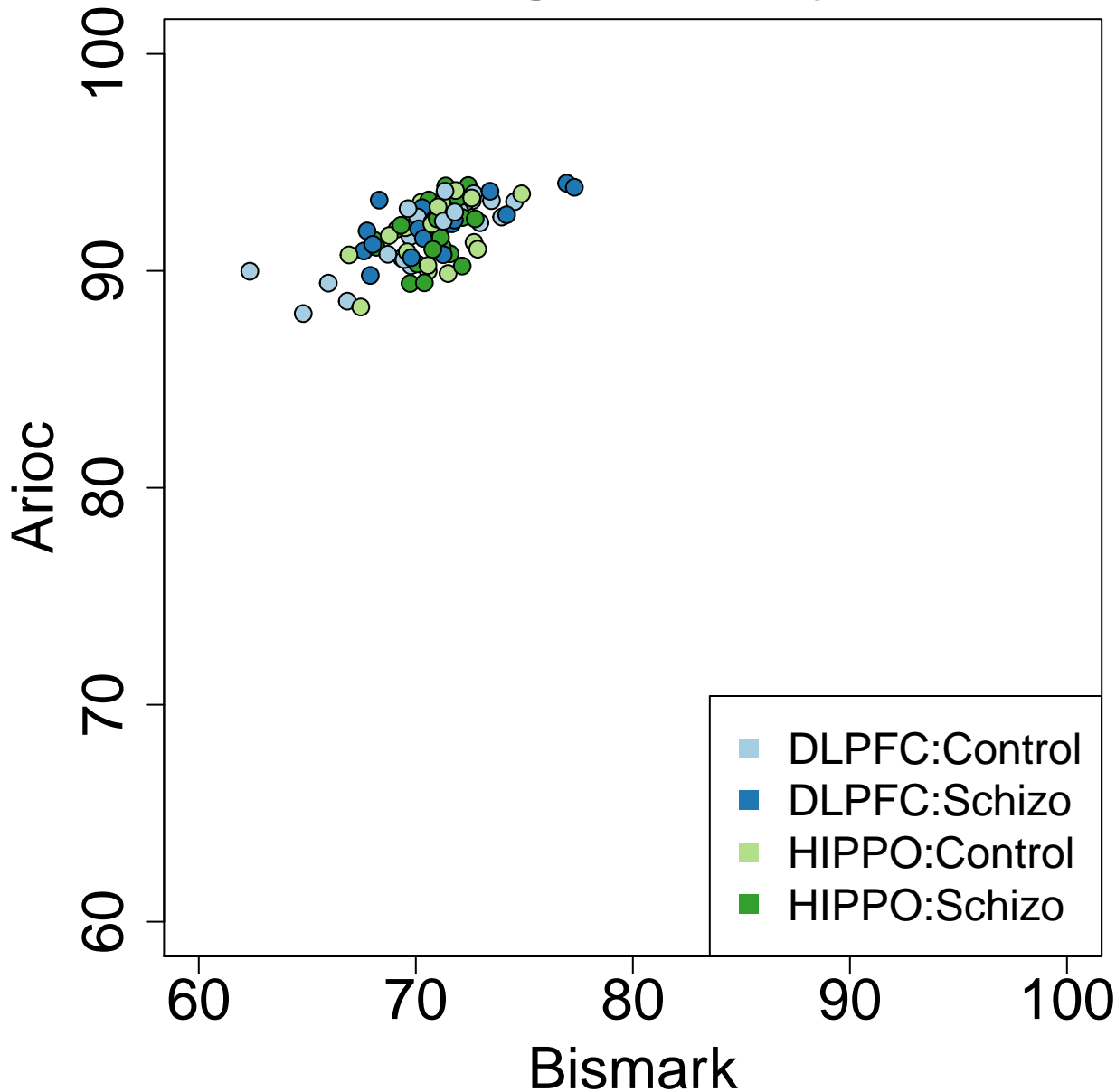

# % CpGs Methylated

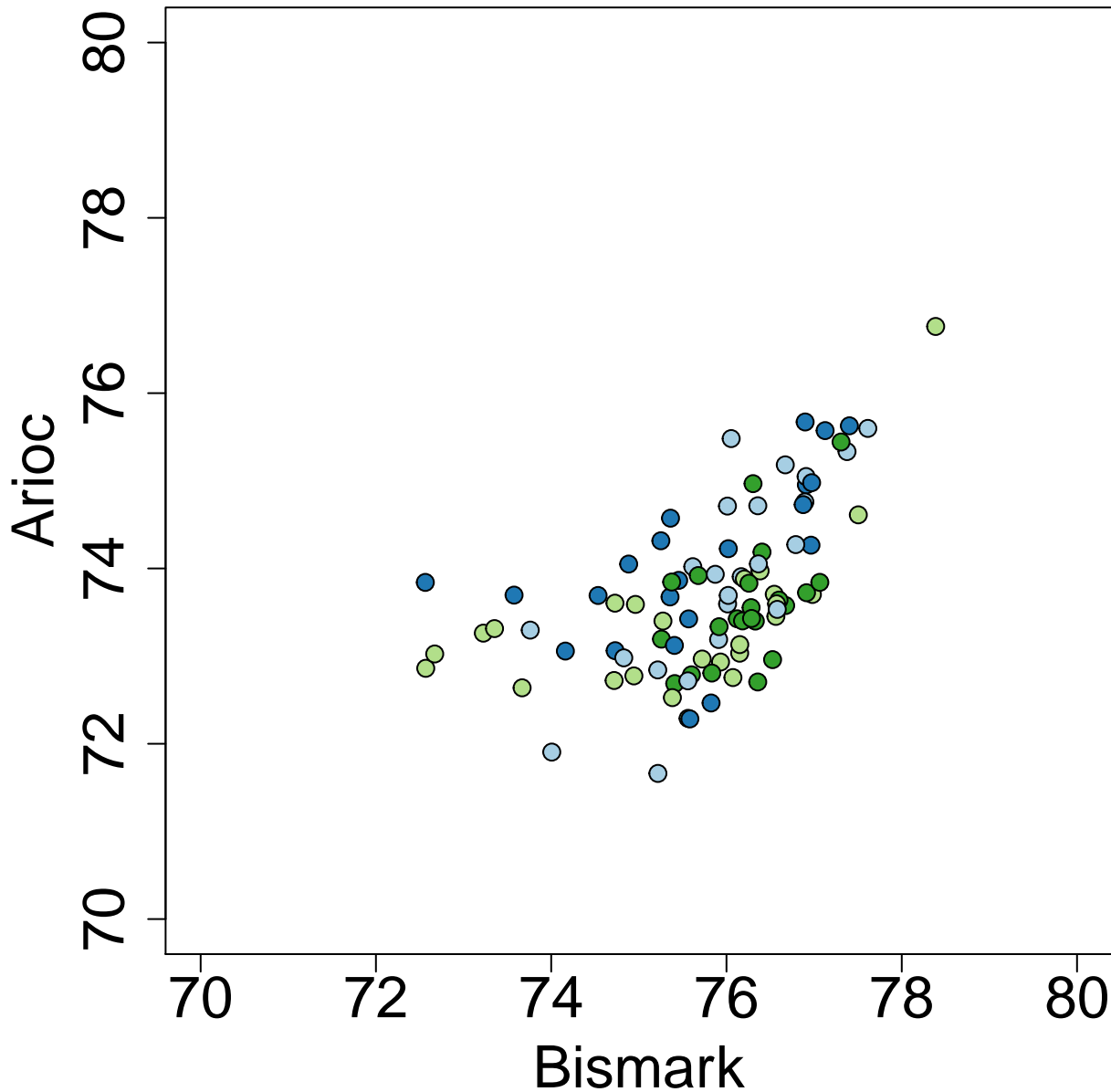

# % CHGs Methylated

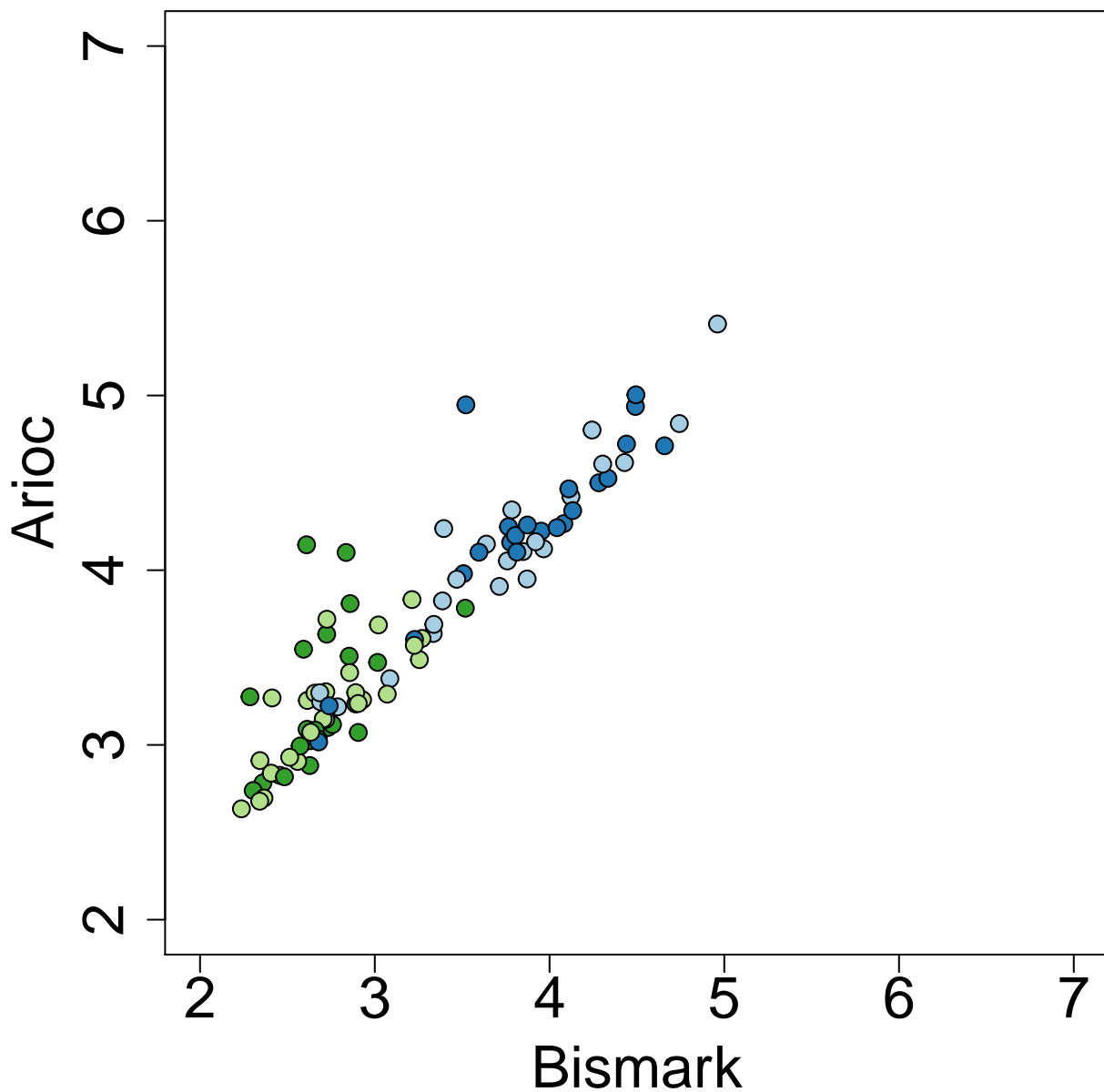

Supplement: Supplementary file 3 — Additional file 3. Comparison of alignment results between Arioc and Bismark. In 94 postmortem human brain samples from the dorsolateral prefrontal cortex (DLPFC) and hippocampus (HIPPO) [40], Arioc 1.25.2401.18201 [8] maps reads at a consistently higher rate than Bismark 0.19.0 [7], while finding similar methylation rates in different cytosine contexts. Results are colored by brain region and diagnosis (control and schizophrenia), but are fairly uniform across these categories, with the exception of generally higher CHG-context methylation reported by both tools in the DLPFC. [file 12859_2023_5461_MOESM3_ESM.pdf]
